# Pyogenic Granuloma in the Mandibular Anterior Gingiva: A Case Study

**DOI:** 10.7759/cureus.52273

**Published:** 2024-01-14

**Authors:** Prasanna R Sonar, Aarati S Panchbhai

**Affiliations:** 1 Oral Medicine and Radiology, Sharad Pawar Dental College and Hospital, Datta Meghe Institute of Higher Education and Research, Wardha, IND; 2 Dentistry, Sharad Pawar Dental College and Hospital, Datta Meghe Institute of Higher Education and Research, Wardha, IND

**Keywords:** gingival pyogenic granuloma, soft tissue enlargement, benign lesion, gingiva, pyogenic granuloma

## Abstract

The oral cavity is frequently affected by gingival pyogenic granuloma (PG), a benign tumor that is known for its quick growth and tendency to hemorrhage. The clinical presentation, diagnostic procedure, and treatment of a patient with gingival pyogenic granuloma are all detailed in this case study. A female individual aged 25 years in otherwise good condition arrived with a sessile gingival tumor in the anterior mandibular region that was expanding quickly. During dental hygiene procedures, there was intermittent bleeding and discomfort related to the lesion. Based on histology analysis and clinical examination, PG was diagnosed. The course of treatment included surgical excision followed by a histological analysis to ensure total eradication. Appointments for follow-up revealed adequate healing and no indications of recurrence. This case study aims to demonstrate the need for prompt diagnosis, appropriate treatment, and diligent follow-up to effectively manage gingival pyogenic granuloma. Dental professionals can better treat patients and achieve better results with a thorough understanding of this common oral lesion and its management.

## Introduction

Many pathologic illnesses can cause oral soft tissue enlargements, making diagnosis challenging in many situations. An expansion can reflect neoplasms, inflammation, cysts, developmental anomalies, and a range of typical anatomic structures. These lesions contain reactive hyperplasias resulting from a recurring, chronic injury that sets off an excessive or exuberant tissue healing response. The most prevalent condition resulting in soft tissue enlargement is pyrogenic granuloma (PG).

Pyogenic granuloma (PG) is the term for a benign vascular origin lesion [1]. Pyogenic granulomas are hyperactive benign inflammatory lesions [2,3]. Since the tumor isn't linked to pus discharge and does not have granuloma-like histology, this term is deemed inaccurate [4-6]. Other names for PG include lobular capillary haemangioma, granulation tissue-type haemangioma, eruptive haemangioma, pregnancy tumor, and granuloma gravidarum [1,4-6]. Although this illness may occur regardless of age, it is more prevalent in the second and third decades of life [1,4-6]. Kadeh found that in patients 30.4 (± 14.9) years old, pyogenic granuloma accounts for 37% of all gingival reactive lesions [7]. It is present on mucous membranes and skin, especially the tongue, cheeks, lips, and gingiva [1,4-6]. Females with elevated steroid hormone levels in their mucosa are typically affected by PG. Most specialists agree that female sex hormones are important in the pathophysiology of this disease [2,3]. It is a growth that resembles a tumor, is usually seen around the anterior teeth, and is thought to be neoplastic in origin [8,9]. It typically arises as a result of multiple triggers, like hormonal fluctuations [10,11], mild local irritation [8,10,11], traumatic injury, or particular medications [12,13]. PG may develop following a hypersensitivity reaction linked to medications such as carbamazepine, phenytoin, nifedipine, levothyroxine, ramucirumab, and calcineurin inhibitors (cyclosporine and tacrolimus). Furthermore, retinoid, antineoplastic, and antiretroviral medications are all related to PG [14]. Trauma accounts for around one-third of lesions; another factor could be inadequate dental hygiene [2,8,10,13]. It usually appears as a sessile or pedunculated swelling of the gingiva, which is asymptomatic [15]. The preferred course of treatment for these lesions is wide surgical resection [16].

This article covers a patient with PG who first presented with a localized development in the mandibular anterior region of the jaw that resembled a malignancy. The etiology, clinical and histological characteristics, and therapy modalities were highlighted in this article, which supported the diagnosis of PG in this case report.

## Case presentation

A female of 25 years old complained of pain during eating due to swelling in the lower front area of her jaw. The patient stated that she became aware of the condition six months prior; during this time, it became progressively larger without any pain. During scaling, there was intermittent bleeding and discomfort related to the lesion. She had stopped brushing in the same region because slight bleeding was present. The patient had no history of systemic conditions.

**Figure 1:**
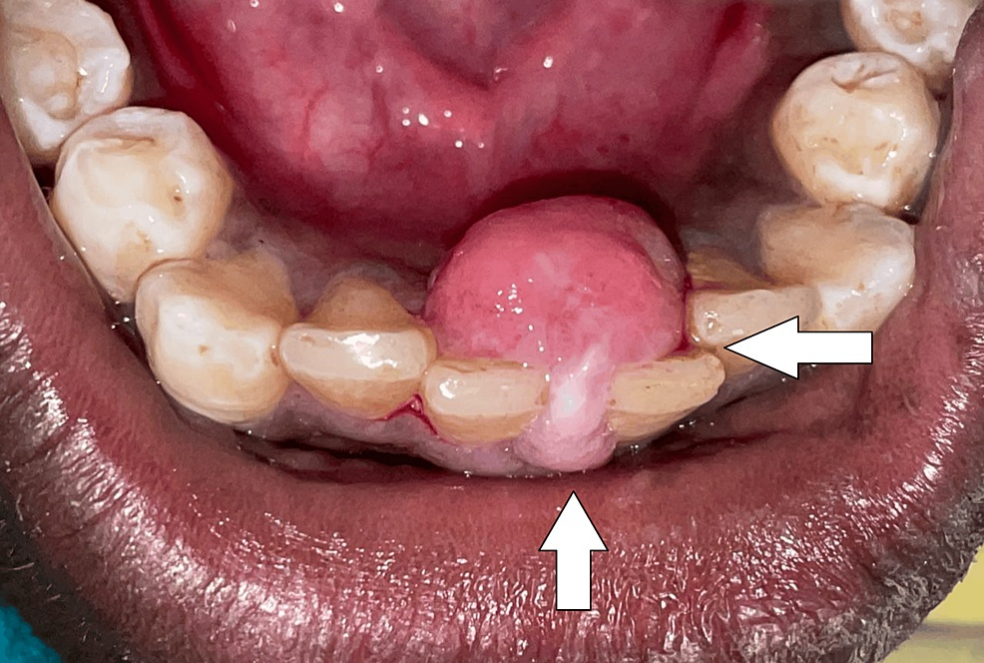
Intraoral swelling in 31, 41 region. Image Credit: Prasanna Sonar

**Figure 2:**
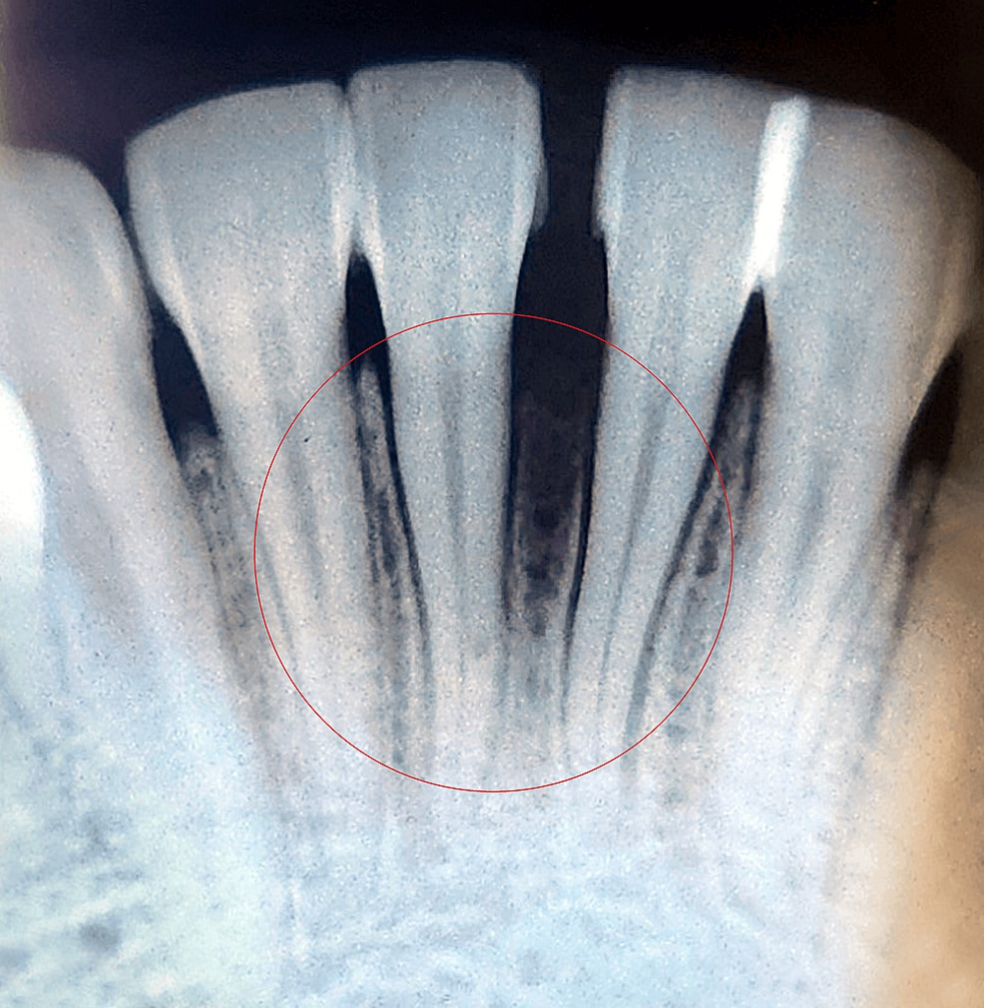
Intraoral periapical radiograph in 31, 41 region. Image Credit: Prasanna Sonar

On extraoral examination, no apparent swelling was present. An intraoral examination revealed a pink, painless, sessile, asymptomatic swelling that was soft on palpation. It had a smooth or lobulated surface and a soft to fibrous consistency with borders well demarcated. Intraoral swelling was extended lingually. Figure 1 depicts the intraoral swelling in 31 and 41 regions. The patient's dental hygiene was poor. The corresponding teeth exhibited no mobility. According to radiography, as Figure 2 illustrates, there was a loss of alveolar bone in the 31, 41 region. The lesion was usually asymptomatic, non-ulcerated, and had a pink or normal coloration. The lesion arises from the connective tissue of the gingiva and often occurs in localized areas. Therefore, gingival fibroma was the tentative diagnosis. The differential diagnoses were given as pyogenic granuloma, peripheral giant cell granuloma, ossifying fibroma, and hemangioma.

**Figure 3:**
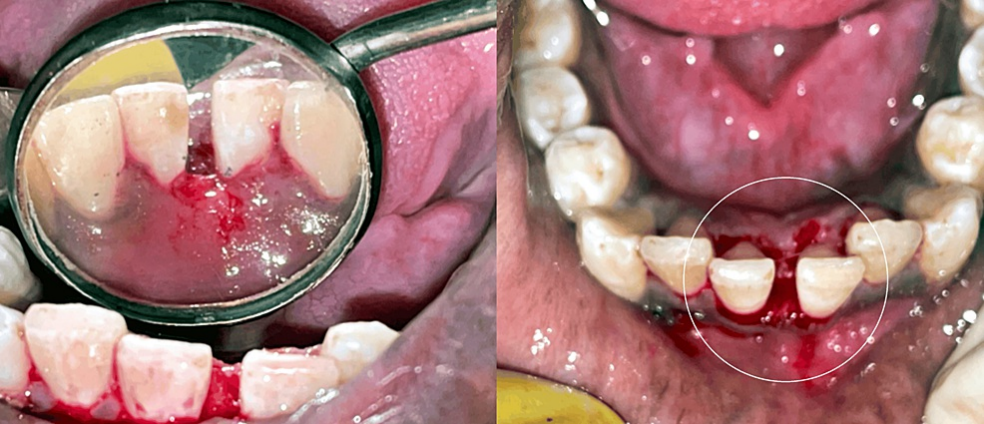
Excision of the lesion. Image credit: Prasanna Sonar

**Figure 4:**
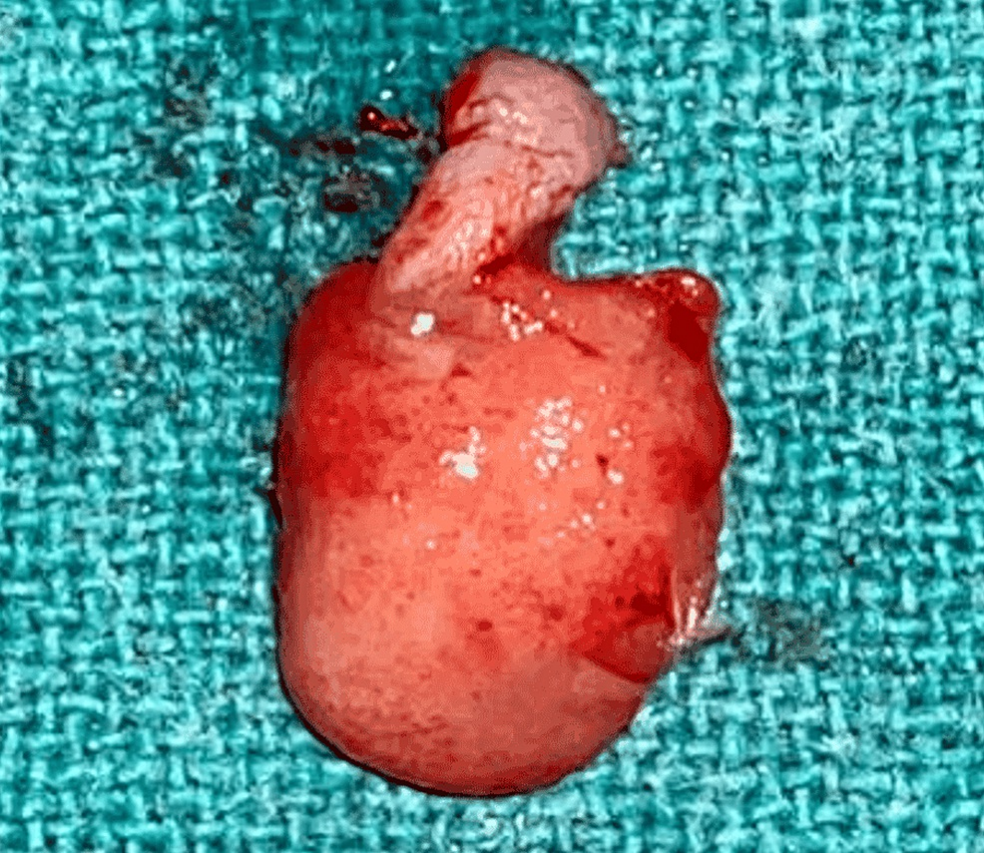
Excised tissue. Image credit: Prasanna Sonar

**Figure 5:**
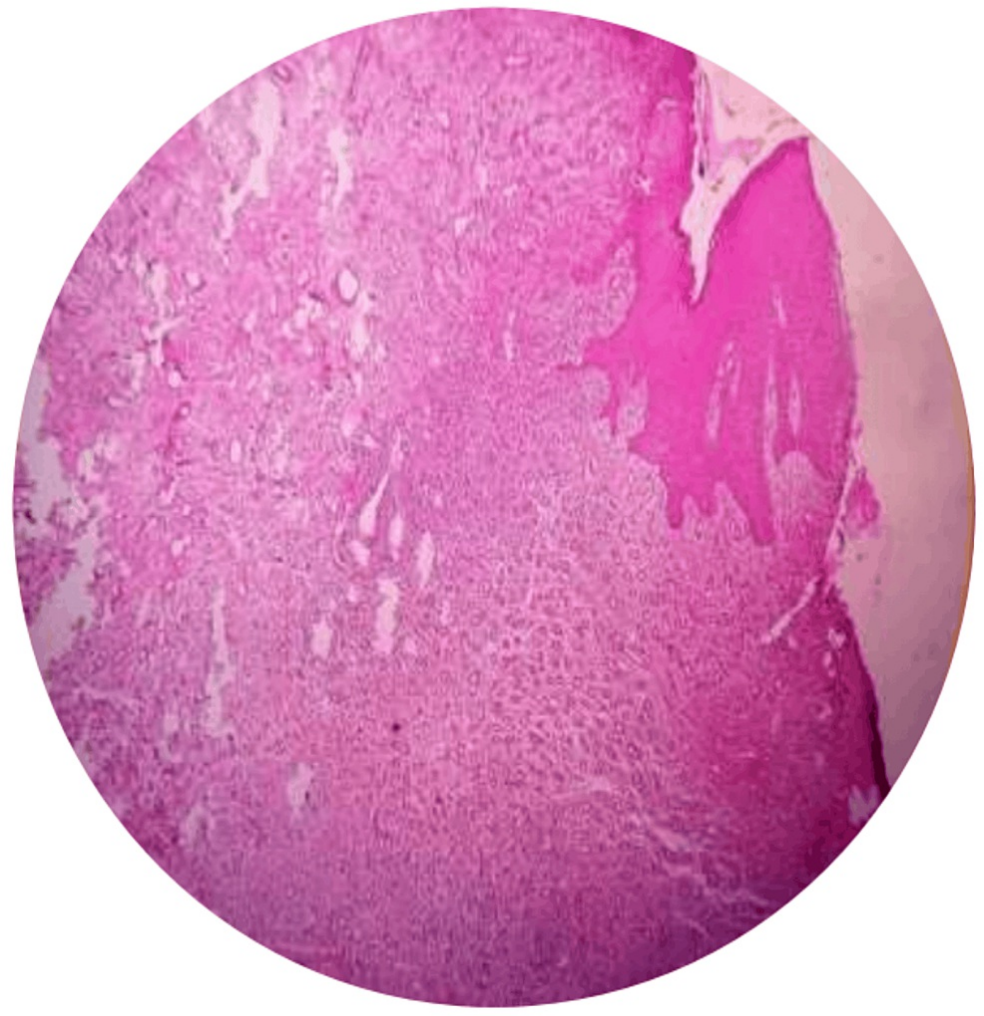
Histopathology (H&E, 10x) of the excised specimen reveals a thick infiltration of inflammatory cells and numerous capillaries embraced with endothelial cells. Image Credit: Prasanna Sonar

**Figure 6:**
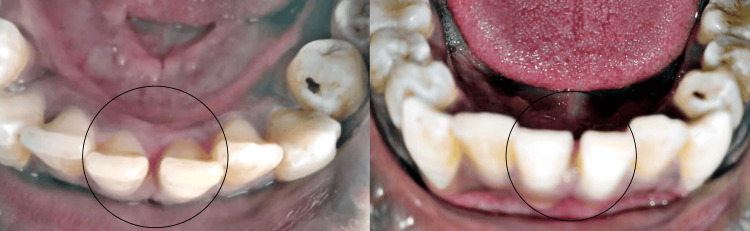
3 months follow-up visit showing no recurrence. Image credit: Prasanna Sonar

A surgical excision was planned for the patient. After finishing the oral prophylaxis, the lesion was removed aseptically. Under local anesthetic, a scalpel and 15C blade were used to surgically remove the lesion, extending up to the mucoperiosteum. The affected teeth were then scaled and curetted. The lesion was excised, as seen in Figure 3. The eradication of aetiological factors lowers the chances of recurrence. The patient was first given information about proper dental hygiene and was encouraged to maintain oral hygiene. A week later, the patient was recalled for examination and for the periodontal dressing to be removed. The removed tissue was submitted for a histopathological analysis, as seen in Figure 4. According to the histopathological analysis, the epithelium was para-keratinized, stretched in certain areas, and grew in the lesion's base. In the connective tissue stroma underneath, there were evident dilated blood vessels, extravasated red blood cells, angiogenesis, a few inflammatory cells, and bundles of collagen fibers. The diagnosis of pyogenic granuloma was confirmed histologically, as shown in Figure 5. Follow-up visits were recommended at one week, one month, and three months. As seen in Figure 6, there was no recurrence after three months. This case will be followed up on for a year to determine whether there is any chance of recurrence.

## Discussion

According to Bhaskar et al.'s research, oral pyogenic granuloma (PG) accounted for about 1.85%, excluding gingivitis and caries [2]. In a study of 244 gingival lesions, oral PG accounted for 52.71% of cases, turning it into the most common lesion. 75.5% of patients had non-neoplastic lesions [17].

It is now universally acknowledged that the genesis of this lesion is an exaggerated response of the local connective tissue to a small injury or any underlying inflammation [18]. Some irritating factors include calculus, inadequate oral hygiene, multiple infections like Staphylococcus aureus infection, overhanging restorations, trauma like pinching the individual's cheek, etc. Hyperplasticity occurs in the underlying fibrovascular connective tissue due to this stimulation caused by irritation, which in turn causes granulation tissue to proliferate and form a PG [19,20]. According to Regezi et al., pyogenic granulomas are believed to be excess connective tissue proliferation in reaction to an injury or known stimuli, like a calculus or foreign material inside a gingival tissue [21]. Several "etiologic factors" were proposed when patients showed symptoms like food impaction, complete periodontitis, trauma, damage to the primary tooth, prolonged irritation, hormones, medications, gingival inflammation, preexisting vascular lesions, chronic irritation from the exfoliation of primary teeth, eruption of permanent teeth, defective fillings in the lesion's region, prolonged irritation from the toothbrush, etc. [22]. Because of the size and location of the lesion, the most likely etiologic factors, in this case, were occlusal interference during eating, calculus from poor oral hygiene practices, and repeated trauma from tooth brushing, causing the lesion's enhanced vascularity. This lesion most likely developed as a result of these circumstances.

Though it can happen at any age, PG is more common in females in their second decade; this may be because of the vascular effects of hormones [16,23]. It is thought to be a hormone-dependent lesion that is most frequently observed following the first trimester of pregnancy. In inflammatory tissues, the high concentration of sexual hormones promotes the synthesis of angiogenic factors. These substances, which are crucial for the development of blood vessels, are found in pyogenic granulomas in high concentrations during pregnancy and lower concentrations after childbirth [4,16]. According to Yuan et al., the process of angiogenesis in oral PG in pregnancy was supported by the greater morphogenetic components in pyogenic granuloma compared to normal gingiva [24]. Granuloma gravidarum, another term for pregnancy tumor, is another term for PG because it frequently affects pregnant women [18].

The tongue, lips, buccal mucosa, and gingiva are the next most often afflicted areas [18]. Clinically, PG is typically observed as an exophytic lesion that is smooth or lobulated with a sessile or pedunculated base. Numerous PGs proliferate, reach enormous proportions, and typically show no symptoms [25]. The growth's vascularity determines the color, ranging from red to pink to reddish purple [26,27]. The gingiva is affected more than the alveolar area, especially the marginal gingiva [9].

There are two histological variants of PG: lobular and nonlobular. The existence of more proliferating blood vessels with little to no particular alterations is what distinguishes the lobular form. The non-lobular form aligns with the endothelial cells and is distinguished by the presence of dilated capillary channels [6]. The fibrous connective tissue frequently exhibits edema. Some cells cause inflammation, such as lymphocytes, plasma cells, or polymorphonuclear neutrophils. An underlying epithelial cuff can be present. [1,27-29] PG has a varied histological appearance due to its inflammatory nature. It can develop into a more mature, collagen-rich, less vascularized structure that eventually becomes a fibrous epulis [1,6]. From a histological perspective, the tumor resembles a granulomatous lesion rather than a pyogenic lesion. But since this phrase is widely used and accepted, any attempt to alter it could cause misunderstandings [4,5,27-30].

Usually, radiographic findings are not present [30,31]. Nonetheless, Angelopoulos [31] concluded that localized alveolar bone resorption was occasionally brought on by long-standing gingival PGs. The radiographic findings in this case indicate alveolar bone loss, as seen in Figure 2.

Treatment recommendations include lesion excision and biopsy unless the lesion might cause a substantial deformity, in which case an incisional biopsy is advised [32]. It is recommended to remove calculus, plaque, and foreign material together with the lesion with cautious surgical excision for mild, painless, non-bleeding lesions. It is recommended to eliminate any visible sources of discomfort by scaling, root-planing, and excising gingival lesions to the periosteum in adjoining teeth [22]. If PG is eradicated along with all contributing reasons and its base, it usually doesn't recur. A surgically treated case of PG is presented in this paper. Scaling and root planning of teeth in the vicinity was done to eliminate any potential local irritants, which may have been the cause of the current case. Several practitioners have employed Nd: YAG laser, sodium tetradecyl sulfate sclerotherapy [17], carbon dioxide laser, cryosurgery, electrodesiccation, intralesional steroids [25], and flash lamp pulse dye laser as additional treatment techniques. Treatment options for pregnancy-related oral pyogenic granulomas can vary based on the individual situation, but they may include preventative measures, including brushing with a soft toothbrush, practicing excellent oral hygiene, and getting rid of dental plaque [33]. Wang advised stopping the bleeding by desiccating the hemorrhages, applying pressure to the lesion, using blood transfusions to stop severe bleeding from a pregnancy tumor, and in extremely rare circumstances, ending the pregnancy owing to uncontrollably high eclampsia [34]. Sometimes, the lesion shrinks after pregnancy, negating the need for surgical intervention [13]. Treatment should ideally be postponed childbirth because oral PGs frequently recur in pregnant women [35]. However, if required, the patient should be managed in the second trimester of pregnancy, and postpartum care can be continued for the patient [33].

## Conclusions

A typical cutaneous and oral cavity lesion, especially to the gingiva, is a pyogenic granuloma. The presentation of this work leads to the conclusion that the inflammatory tissue may have crossed the line from normal gingivitis to the production of granulomas due to a combination of several etiological variables. This case report reminds dental and medical professionals of the importance of identifying and treating gingival pyogenic granuloma properly. Positive results can be achieved by using a multidisciplinary strategy that includes precise diagnosis, individualized treatment plans, and patient education. For individuals affected, this will improve their overall health and oral health.
